# The Use of Conservative and Alternative Therapy for Low Back Pain

**DOI:** 10.3390/medicines2030287

**Published:** 2015-09-09

**Authors:** Ping Chung Leung

**Affiliations:** 1 State Key Laboratory of Phytochemistry and Plant Resources in West China, Kunming 650201, Yunnan, China; E-Mail: pingcleung@cuhk.edu.hk; Tel.: +852-2252-8868; Fax: +852-2632-5441; 2 Department of Orthopaedics and Traumatology, Institute of Chinese Medicine, the Chinese University of Hong Kong, Shatin, N.T., Hong Kong, China

**Keywords:** low back pain, treatment, alternative treatment, natural healing

## Abstract

Low back pain may have complex patho-physiological causes leading to chronicity that resists conventional managements. Complementary and alternative treatment options have, therefore, gained popularity. In this chapter, acupuncture, manual therapy, and natural healing for low back pain will be discussed. Special emphasis is given on the role of the individual in the control and prevention of low back pain.

## 1. Introduction

Millions of people in the world suffer from low back pain (LBP), both in the acute and chronic situations. Acute onsets commonly become chronic and chronic conditions may be complicated with acute exacerbations [1]. Chronic low back pain is one of the most frequently experienced neuromuscular ailments in all communities. In Europe and America, 80% of adults experience significant back pain during their lifetime [2,3]. It is estimated that 10% to 20% of affected adults develop symptoms of chronic lower back pain [4]. There are many causes of LBP some of which have definite pathology, like spinal injuries, infection, degenerative arthritis and tumors. Others have obscure pathology not revealed even after long clinical courses. Presumably early or preclinical stages of pathological changes might remain obscure even after standard clinical investigations [5,6,7].

Since LBP is such a common condition, many specialists in many disciplines of the current medical practice have close collaborations. Different disciplines have their own areas of special concern. Thus, orthopedic experts would concentrate on the bony spine and intervertebral discs. Neurosurgeons are more concerned with the intervertebral nerves and spinal cord. Generalists usually take a less committed attitude. With the advent of Computed Tomography and Nuclear Magnetic Resonance Imaging both bony structures and soft tissue shadows are shown much more clearly so that early presentations of common pathologies become better revealed. However, the correlation between the actual clinical presentations and the structural changes in the images could be poorly matched. For a desperate LBP patient and his physician or surgeon who is eager to help, although the clinical pictures might not be well-matched with pathological images, active interventions, mostly of a surgical nature, might still be the treatment offer, failing to arrive at an ideal outcome [8,9].

## 2. Common Causes of LBP

The detection of diseased intervertebral discs with different degrees of “disc herniations” must be a very common finding in a MRI examination. An early bulge could be taken as the culprit of an attack of LBP, hence taken as an indication for surgical intervention. Yet, we know that such picture of disc bulging occurs not infrequently among young and middle-aged people who are not suffering from LBP at all. One cannot be certain whether surgery is really indicated for just bulging discs. On the other hand, acute pain turning chronic, whether related or unrelated to bulging discs in spinal imaging, are not unusual [10,11].

As one studies the complex structure of the spine which involves not only the bony vertebral column with its multiple components and neurological tissues, inside and through-out, but also the closely related enwrapping muscles, one should realize that many other possible causative factors could be responsible for the acute and chronic pain [12].

The known pathologies possibly causing LBP are:

*Lumbosacral Strain* is most common among manual laborers who need to carry heavy loads. The pain is located generally around the lumbosacral region without any preference to either left or right. Investigations including modern imaging often give negative results. The high frequency of this condition related to worker’s compensation has led to a common interpretation of socio-psychological orientation [13]. Probably because of this background, studies on LBP in the last decades have excluded cases of worker’s compensations [14].

*Posterior Facet Syndrome* refers to the early degenerative arthritis of the posterior facet joints which suffer from loss of motion, thus causing stiffness of the whole spine and LBP [8]. This could be the commonest cases of LBP involving the elderly [15,16].

*Muscle/Sacroiliac Syndrome* refers to stiffness and spasm of a specific or a group of paraspinal muscles. The typical presentations are general ache related to the particular muscle involved [17,18].

*Spondylolithesis* may be a harmless in-born structural anomaly if it is mild (degree 1 and 2). Otherwise the unusual mobility at the site of spondylolithesis could be the cause of lumbosacral strain or paraspinal muscle syndrome [19,20].

*Osteoporosis* affecting the vertebral bodies leading to gradual collapse and pain is common among the elderly. With the compression collapses of the vertebral bodies, the intervertebral discs could often be identified as bulging and herniating. In fact, the LBP will gradually go away as the trabecular long compression gets consolidated with time [21,22].

When the listed causes of LBP are coupled with varying degrees of evidences of some other common pathologies, like disc herniation, and especially when the pain turns chronic, surgical interventions could be a common outcome, although supporting evidence might not be sufficient [23,24].

Apart from the common causes of LBP described, other causes include sacroiliac strain, spasm of the piriformis muscle or psoas muscle, which may all present as lumbago without any specific symptomatology.

## 3. Complementary/Alternative Treatment for Low Back Pain

### 3.1. Acupuncture

When evidence in support of drastic treatment like surgical intervention are insufficient, complementary/alternative treatment for LBP could be considered. The majority of chronic back pain patients do not have diagnostic evidence of pathology that deserves special medication or surgery [25].

In spite of energetic rehabilitation training, no more than 50% of patients had full recoveries [26]. Electrical stimulation for control of pain is often employed, which later develop as transcutaneous electrical nerve stimulation (TENS) [26]. Since the endorsement of acupuncture treatment as an effective option for pain control in the US, acupuncture has become popular [27].

Coan reported that patients receiving a minimum of eight acupuncture treatments experienced less pain at a 40-week follow-up [28]. Lehmann, *et al.*, conducted a trial to assess the efficacy of TENS and electro-acupuncture (EAP) in the rehabilitation of chronic back pain patients of the fifty-four patients treated in a three-week inpatient rehabilitation program. The EAP group consistently demonstrated greater improvement on the outcome measures than the other treatment groups [29].

A recent Cochrane review of 35 trials, evaluating the effectiveness of acupuncture for treatment of non-specific LBP and dry-needling for myofascial pain syndrome in the low-back region compared to no treatment, sham therapies, and the addition of acupuncture to other therapies, provided further evidence on the effectiveness of acupuncture for chronic LBP [30]. The evidence suggested that acupuncture is more useful for pain relief and functional improvement when used in conjunction with other conventional therapies than either conventional therapies or acupuncture alone. The overall observation was that acupuncture may be useful as either a unique therapy for chronic LBP or an adjunct therapy to other conventional treatments.

Although the effects of acupuncture on pain control is obviously less effective than the use of analgesics that brings immediate, short-term relief, it has great practical value as an alternative for chronic persistent pain [31,32,33]. Recent studies using functional MRI indicated that specific regions of the brain could be activated during procedures of acupuncture [34]. Acupuncture has been the most popular form of complementary, alternative treatment in modern hospitals and clinics, especially among pain teams [35] and expectedly, will become more accepted in the following days.

As acupuncture has become common hospital practice and among physiotherapists, discussions have started on the choice of acupoints along the meridians: what are the principles behind the selection of acupoints for the treatment of chronic LBP? The traditional Chinese practitioners would choose according to observed “syndrome” complexes taken to be related to loss of internal balance, e.g., “renal insufficiency” or “circulatory stagnation”. Practitioners with neurological knowledge would choose according to the detection of neurological involvement of the intervertebral nerves. The use of a large number of puncture needles in contrast to a selected number is another point of argument among the advocates. Chinese acupuncturists have seriously engaged in clinical practices in order to find a proper answer to the controversies. While research is still ongoing, the answer is still obscure. At this moment, perhaps the use of a limited number of acupuncture points according to the root levels of pain presentation could be a good practical choice [33].

### 3.2. Manipulative Treatment

The indications for manipulation in patients with LBP are as follows:

● Uncomplicated low back pain (lumbago)
● Sciatica without neurological deficit
● Uncomplicated chronic low back pain
● Post-surgical chronic low back pain
● Intervertebral disc degeneration
● Posterior facet syndrome
● Sacroiliac syndrome
● Sacroiliac strain
● Piriformis syndrome
● Psoas syndrome
● Spondylolisthesis Grade 1,2

Valuable information concerning manipulations are available in old literature. One report which compared the results of Spinal Manipulative Therapy (SMT) in patients with acute and chronic back pain was presented by Potter [36]. His conclusion, that chronic pain was less responsive than acute pain, is not surprising. He did, however, claim that 71% of patients classified as having uncomplicated chronic LBP showed improvement of their symptoms. Similarly, Riches claimed that 86% of patients diagnosed as having chronic back strain improved following manipulation [37].

Acute low back pain complicated by sciatica or neurological deficit responded equally well to manipulations. Potter found that chronic leg pain improved in approximately 70% of patients treated with SMT; the number of successes decreased considerably when neurological signs were present [36,37,38].

Cyriax felt that the primary effect of manipulation was the reduction of disc protrusions [39], although his expectation has never been confirmed with modern imaging techniques. On the other hand, Kirkaldy-Willis and Cassidy were unimpressed with the results of manipulation in patients with demonstrable nucleus pulposis herniation [40].

For posterior facet syndrome, there is an increase in the mobility of the spine following manipulation. Some of the best results from spinal manipulation have been reported in patients where the diagnosis of sacroiliac syndrome has been made. Over 90% of patients with this diagnosis show improvement in their back pain following manipulation [41]. Experts on manipulation place a great deal of emphasis on the analysis of sacroiliac motion and position and choose their manipulation technique according to these findings. They believed that manipulation is the most effective method of treatment for sacroiliac syndrome [42].

Specific manipulation techniques have been developed with the aim of stretching or manually massaging the spinal muscles in an attempt to relax them, although the importance of the muscle syndromes in the genesis of back pain remains speculative.

Back pain associated with spondylolisthesis was found to improve through spinal manipulation in 85% of cases reviewed by Kirkaldy-Willis and Cassidy [43]. These experts made it quite clear that no claim should be made on any structural correction. Instead, they speculated that in most cases, the spondylolisthesis could be an incidental finding.

### 3.3. Natural Healing

For all chronic conditions, in spite of the most energetic attempts to heal, the problem often remains, often resulting in more and more drastic offers from the attending clinicians. Alternatively the victims of chronic pain need to try, successfully or unsuccessfully, to live with their misery.

Oriental medicine has proposed a concept of natural healing which could be useful for the chronic sufferers.

One of the mostly emphasized areas of health in the earliest classics of Chinese medicine, Ne-jing, is Natural Healing: the way to maintain a perfect harmonious state of physical, physiological, and psychosocial well-being. The overall concept involves the harmony between *yin* and *yang*; harmony between physique and psychosocial state; balanced nutrition, balanced exercises, and recreation, which are considered all interlinked [44,45,46].

Natural Healing is understood as a treatment program directing against a specific target in the rehabilitation process in Europe and America. Natural Healing in the Chinese Medicine context has a much broader conceptual meaning, which includes maintenance of health, wellness and prevention of falling sick. Many practical systems of Natural Healing are advocated. The best known include *Tai Chi* and *Qi Gong*, which demand rigid systems of chained physical activities [47]. When the contents of the chained physical activities are analyzed, three major components become obvious.

#### 3.3.1. Stretching

Spiritual dancing could have been the very early practice of Natural Healing. Stretching movements in a variety of postures have become the most essential components of practice. Imitation of animal movements evolved into sophisticated chains of stretching activities. Later, different groups of practitioners created their own systems of stretching, with different connotations and emphases. One uniform component of these different systems is that all of them consist of slow stretching movements. As far as posture is concerned, some advocate natural postures like standing, modification like “Buddha sitting”, half-kneeling, *etc.* All activities demand a gradual stretch of muscles, tendons, and joints to their extreme limits.

#### 3.3.2. Respiratory Control

Natural Healing includes practice of controlled breathing which goes along with the stretching exercises. *Qi Gong* might be mistaken as controlled respiration only. In fact, it requires simultaneous stretching, controlled respiration, and meditation. It is believed that, with the skillful control of breathing, *qi* is successfully manipulated, so that it not only circulates through the respiratory system, but, together with meditation, it reaches out to the other physiological systems to improve their metabolic state of balance. What is “qi”? Literally, “qi” means respiration, but in the philosophy of Chinese medicine “qi” referred to a state of bodily harmony based on a perfect oxygenated state, together with a peaceful mind.

Respiration is controlled to follow an unusual inspiration/expiration pattern, which means extra-long inspiration or extra-long expiration. Abdominal or diaphragmatic breathing is also practiced. While doing so, the pelvic diaphragm and anal sphincters are also squeezed at will.

In short, respiratory control is executed simultaneously with the stretching movements in a smooth synchronized chain of activities under the individual’s free will. The individual could develop his/her own policy of training which could be amended from time to time [48].

#### 3.3.3. Meditation

Natural Healing needs to achieve harmonizing physical, humoral, and mental activities of which meditation is an indispensible component. The skillful practitioner attains a tranquility of the mind while stretching is being performed with controlled breathing. The apparently complicated system of movements in *Tai Chi* should not be hindering meditation. Rather, they provide a good initiating environment where the day to day mental pressure will not be felt. The material background for meditation is resting of mental activities so that the brain is freed from motor and sensory burdens, relieved from complex memories, protected from emotions and problem solving requirements. With this unchallenged mental state, a reorganization of the interacting neurological messages can take place, initiating a state of harmony [49].

Unlike Natural Healing in Europe and US, which have specific demands and needs, Natural Healing in the Orient is more of a self-promotion of wellness and longevity. We might not feel particularly threatened by the imagination of a special disease, but we certainly do not want to fall sick. Since the tripartite practice of stretching, controlled breathing, and meditation can be easily learned, comfortably practiced, and freely modified, the practice could gain more popularity.

In fact the same requirements, *viz*. stretching, controlled breathing and meditation, are also required in the popular health promotion exercise in India: the yoga practice. Stretching undoubtedly stimulates the proprioceptive receptors involved which are responsible for the activation of the pain control neurological activities described within the “Gate theory” [50].

#### 3.3.4. Anatomical and Physiological Basis of Stretching in Relation to Pain Control

As discussed earlier, LBP has multiple causes related to the bones and joints, the intervertebral discs as well as the structure around the spine. The spine is closely enwrapped by the paraspinal muscles. Many outlying tissues are intimately involved in motion when the spine moves. Tissues include joint capsules, ligaments, tendons, aponeuroses, retinaculae, and intramuscular fasciae: all these are connected through a network of connective tissues called fascia. The fascia has been taken as insignificant anatomical tissue that possesses no special structural or physiological role except that it fills up inter-tissue spaces. Recent studies have overturned the old concept. The fasciae contain nerves and proprioceptive receptors as well as small vessels. The widest possible network of soft tissues is formed by the fasciae which unite the groups of neuro-vascular tissues, muscles, bones and joints, and even the internal organs [51]. The network of soft tissues allows the free gliding of adjacent structures, as well as helping the transfer of forces between them. Neurologically, the stimulation of the proprioceptive receptors initiate coordinating messages to the joints and muscles involved. Mechanically, the interconnections between the separate muscles and muscle groups help to maintain an ideal sharing of loading and stretching activities. On the cardio-vascular side, it has been a well-established fact that limb muscle contractions constitute a “second cardiac pump” [52].

The lumbar fascia has a special role in the situation of low back pain.

Although the pain generating role of intervertebral disc prolapse is undisputed for some cases of low back pain, they might not play a decisive role in the majority of cases. Comparisons of lumbar spine MRI between back pain patients and normal patients have revealed that many people without back pain also show severe disc damages, while many other back pain patients show better discs than pain-free people of the same age [8]. New explanatory models for the generation of low back pain are now recognized: from Panjabi’s proposal that repetitive stress in spinal ligaments and facet joint capsules could induce dysfunction of muscle control which may lead to chronic pain and neural inflammation [4], to pain-related fear inducing a cycle of decreased movement, connective tissue remodeling of lumbar fascial tissues, leading to more inflammation, nervous system sensitization, and more pain. Mechanical stimulation, such as physical therapy, massage, chiropractic manipulation, acupuncture, or by changing specific movement patterns (e.g., movement therapies, yoga), and most notably stretching, may help to reverse the abnormalities.

In a recent study, 109 low back pain patients were randomly divided into four treatment groups. Group 1 was treated with core stability exercises; group 2 conducted the same exercises together with deep breathing; group 3 was treated with myofascial release therapy; and group 4 received the same treatment as group 3 together with deep breathing. Results showed that their myofascial release treatment in combination with deep breathing training led to greater improvements than traditional conventional care for low back pain under almost all measures. An increase in parasympathetic tone through manual therapy and breathing was suggested as a possible mechanism by the authors [53]. Patients should realize that myofascial release can be self-administered through repetitive slow stretching activities.

## 4. Conclusions

While acute unresolving LBP is most likely the result of specific pathologies like acute injuries or disc degenerative changes that deserve specific managements, like surgery, as LBP turns chronic, a complexity of pathological changes contribute towards the lengthy sufferings of the patient.

Pain is the most common health problem for which adults using complementary and alternative managements. Many people with chronic pain conditions turn to these practices to supplement conventional clinical treatment which they are already receiving.

Manual therapies in various forms have enjoyed centuries of popularity, although scientific evidence remains obscure. Acupuncture has emerged as another popular option of treatment in spite of its obscure nature. Despite the widespread use of complementary health practices for chronic pain, scientific evidence on efficacy and mechanisms of action—whether the therapies genuinely help relieve the clinical symptoms and, if so, how—is, in most cases, limited. However, the evidence base is growing, especially for several complementary health practices most commonly used by people to lessen pain. The recent research on the fascia has offered exciting thoughts on the origin and control of LBP. Although more research efforts would be required to add more scientific information to the physiological value of the fascia, the logical observations and rediscovery of the important role of this apparently insignificant interstitial tissue complex, have given extra support to the individual’s responsibility in the healing of his/her chronic LBP [54,55].
